# Hypermethylation of the *hTERT* promoter inhibits the expression of telomerase activity in normal oral fibroblasts and senescent normal oral keratinocytes

**DOI:** 10.1038/sj.bjc.6601291

**Published:** 2003-10-14

**Authors:** K-H Shin, M K Kang, E Dicterow, N-H Park

**Affiliations:** 1 School of Dentistry, University of California, Los Angeles, CA, USA; 2 Jonsson Comprehensive Cancer Center, University of California, Los Angeles, CA, USA

**Keywords:** hTERT, hypermethylation, senescence, fibroblasts, keratinocytes

## Abstract

Telomerase activity in human cells closely correlates with the expression of its catalytic subunit, telomerase reverse transcriptase (*hTERT*). Previously, we reported the lack of telomerase activity in normal human oral fibroblasts (NHOF) and the diminution of telomerase activity during senescence in normal human oral keratinocytes (NHOK). To investigate the underlying mechanisms of telomerase regulation in both cell types, we analysed the expression, promoter activity, and methylation status of the *hTERT* gene. The expression of *hTERT* mRNA diminished in senescent NHOK, but was not detected in NHOF at any stage of replication. An exogenous *hTERT* promoter was active in NHOF and in senescing NHOK, indicating that the lack of *hTERT* gene expression resulted from alteration of the endogenous *hTERT* promoter. Since methylation is involved in the silencing of numerous genes, we carried out DNA methylation assays. The assay revealed that the *hTERT* promoter was hypermethylated in NHOF and was gradually methylated during senescence in NHOK. Treatment of NHOF and senescent NHOK with the demethylating agent 5-aza-2′-deoxycytidine restored the expression of endogenous *hTERT* mRNA. Our results suggest that hypermethylation of the *hTERT* promoter plays a critical role in the negative regulation of telomerase activity in normal human oral cells.

Telomerase is complex, composed of a ribonucleoprotein, reverse transcriptase catalytic subunit, *hTERT*, template, *hTR*, and several associated protein cofactors (Feng *et al*, 1995; Harrington *et al*, 1997; Meyerson *et al*, 1997; Nakamura *et al*, 1997). While *hTERT* is not expressed in most normal human somatic tissues, *hTR* is expressed constitutively in most human tissues (Kim *et al*, 1994; Shay and Bacchetti, 1997). In normal human cells and in tumour cells, telomerase activity is positively correlated with the expression of *hTERT* (Kim *et al*, 1994; Shay and Bacchetti, 1997). Therefore, regulation of *hTERT* expression appears to control telomerase activity. The *hTERT* promoter region has been cloned and characterised (Horikawa *et al*, 1999; Takakura *et al*, 1999), permitting analysis of the molecular mechanisms involved in its regulation (Cong *et al*, 1999; Horikawa *et al*, 1999; Takakura *et al*, 1999; Wick *et al*, 1999). Transcription factors c-myc, Sp1, AP2, AP4, and c-myb act as activators and several repressors, such as WT1, p53, MZF-2, and Mad1, have been identified (reviewed by Poole *et al*, 2001). Analysis of the *hTERT* promoter region also revealed the presence of CpG islands containing CG-rich methylation sites (Cong *et al*, 1999; Horikawa *et al*, 1999; Takakura *et al*, 1999; Wick *et al*, 1999). Promoter region hypermethylation associated with transcriptional loss is an alternative mode of gene silencing for several tumour-suppressor genes (Zingg and Jones, 1997; Baylin *et al*, 1998; Herman, 1999). The hypermethylation status of the *hTERT* promoter has been examined in a variety of human normal and cancer cells (Devereux *et al*, 1999; Dessain *et al*, 2000; Guilleret *et al*, 2002). In these studies, *hTERT* promoter methylation was observed in telomerase-negative, as well as in telomerase-positive cells, suggesting different modes of regulation of *hTERT* expression in methylation-dependent and -independent pathways.

Previously, we reported that replicating normal human oral keratinocytes (NHOK) expressed telomerase activity, which gradually disappeared during senescence, and that normal human oral fibroblasts (NHOF) lacked telomerase activity at all stages of replication (Kang *et al*, 1998). To investigate the mechanism of telomerase inactivation in NHOK and NHOF, we analysed the changes in *hTERT* expression, *hTERT* promoter activity, and methylation status of the *hTERT* promoter, in serially subcultured NHOK and in replicating NHOF. In our present study, we found that the inactivity of telomerase in NHOF and during NHOK senescence was not caused by absence or defective necessary cofactors, but by hypermethylation of CpG islands in the *hTERT* gene promoter.

## MATERIALS AND METHODS

### Cells and culture conditions

Primary cultures of NHOF were established from explants of the gingival connective tissue excised from patients undergoing oral surgery. The cells that proliferated outwardly from the explant culture were continuously cultured in 100-mm culture dishes, in DMEM/medium 199 (4 : 1) containing foetal bovine serum (Gemini Bioproducts, Calabasas, CA, USA), and gentamicin (50 *μ*g ml^−1^). Primary NHOK were prepared from separated epithelial tissue, and serially subcultured in keratinocyte growth medium (KGM, Clonetics, Charlotte, NC, USA) containing 0.15 mM Ca^2+^, as described previously (Kang *et al*, 1998). Adenovirus transformed human embryonic kidney cells 293, obtained from the American Type Culture Collection (Manassas, VA, USA), were cultured in DMEM/medium 199 (4 : 1) containing foetal bovine serum (Gemini Bioproducts) and gentamicin (50 *μ*g ml^−1^). SCC-4 (human squamous cell carcinoma cell line) was obtained from American Type Culture Collection (Manassas, VA, USA), and cultured in DMEM/Ham's F-12 containing 10% foetal bovine serum (Gemini Bioproducts) and gentamicin (50 *μ*g ml^−1^).

Cell population doublings (PD) were determined after subculturing to postmitotic stage. At the end of each passage, PD was calculated by the formulation, 2*^N^*=(Cf/Ci), where *N* denotes PD, Cf the total cell number harvested at the end of a passage, and Ci the total cell number of attached cells at seeding. The PD time was calculated by dividing the duration of culture in hours by the number of PD.

### Analysis of telomerase activity

Cellular extracts were prepared by using CHAPS (lysis buffer) provided from the TRAP-eze Telomerase Detection Kit (Intergen Norcross, GA, USA), as recommended by the manufacturer. Telomerase activity was determined using the TRAP-eze Telomerase Detection Kit as described previously (Kang *et al*, 1998). Each TRAP reaction contained a cellular extract equivalent to 1 *μ*g protein. The PCR products were electrophoresed in 12.5% nondenaturing polyacrylamide gels, and the radioactive signals were detected by PhosphorImage (Molecular Dynamix, Sunnyvale, CA, USA).

### Analysis of endogenous *hTERT* expression

Total RNA was isolated from the cells using Trizol™ reagent (Invitrogen Carlsbad, CA, USA) and further purified through RNeasy columns (Qiagen, Chatsworth, CA, USA). Following isolated RNA solution in 7.5 *μ*l H_2_O, the reverse transcription (RT) reaction was performed in first-strand buffer (Invitrogen) containing 200 U Superscript II (Invitrogen), 40 U RNase inhibitor (Perkin-Elmer, Foster City, CA, USA), 10 *μ*M dithiothreitol, 250 ng random hexamer (Perkin-Elmer), and 2.5 *μ*M dNTP. The annealing reaction was carried out for 10 min at 25°C, and cDNA synthesis was performed for 50 min at 42°C, followed by 10 min incubation at 70°C for enzyme inactivation.

To amplify *hTERT* cDNA, PCR reaction was performed with 1 *μ*l RT product using the primers 5′-GCCTGAGCTGTACTTTGTCAA-3′ (forward) and 5′-CGCAAACAGCT TGTTCTCCATGTC-3′ (reverse). The primer set amplified *hTERT* mRNA species encoding functional full-length (457 bp) or defective spliced isoforms (421, 275, or 239 bp) (Ding *et al*, 2002). PCR products were separated in 2% agarose gels. The *hTERT* signals were further amplified by Southern hybridisation using ^32^P-[dCTP]-radiolabelled probe synthesised from *hTERT* cDNA, as described in detail previously (Kang *et al*, 2002).

### Analysis of *hTERT* promoter activity

A pGL3B-TRTP containing a 1670 bp fragment (−1665 to 5) of full-length *hTERT* promoter upstream of the firefly luciferase gene in the pGL3-basic (Promega Madiso, WI, USA), kindly provided by Dr J Carl Barrett (National Institute of Environmental Health Science), was used for the *hTERT* promoter assay. Prior to transfection, a six-well plate with approximately 5 × 10^4^ cells per well was inoculated and cultured for 24 h. The pGL3-basic (2 *μ*g well^−1^) or the pGL3B-TRTP vectors (2 *μ*g well^−1^) were transfected using Lipofectin reagent (BRL Life Technologies, Inc., Gaithersberg, MD, USA). For better comparison among cells with different transfection efficiencies, the pGL3-control plasmid (2 *μ*g well^−1^; Promega), which has the firefly luciferase gene under the transcriptional control of SV40 enhancer/promoter, was also transfected into each cell and used for normalisation of the activities shown by the pGL3B-TRTP construct. Cells were collected 48 h after transfection and cell lysates prepared according to Promega's instruction manual. Luciferase activity was measured using a luminometer (Promega).

### Methylation analysis of *hTERT* promoter

Methylation of the *hTERT* promoter was studied using methylation-specific PCR with bisulphite-modified DNA and 5-aza-2′-deoxycytidine (5-aza-CdR) treatment (Dessain *et al*, 2000). For methylation-specific PCR with bisulphite-modified DNA, genomic DNAs from cells were isolated using Qiagen Tissue/Blood Kit (Qiagen), and bisulphite-modified according to published methods (Dessain *et al*, 2000). Promoter regions of *hTERT* were amplified from the nonmodified and the modified DNA. PCR reaction products were electrophoresed in 2% agarose and amplified DNA fragments were directly visualised under UV illumination.

Cells were incubated in the culture medium with 10 *μ*M 5-aza-CdR for 24 h on days 3 and 5, and then harvested on day 7 for analysis. Total RNA was prepared using Trizol™ reagent (Invitrogen). Randomly primed cDNAs were reverse-transcribed and *hTERT* cDNA was amplified as described above.

## RESULTS

### Correlation between the expression of endogenous hTERT and telomerase activity in NHOF and NHOK

**Figure 1:**
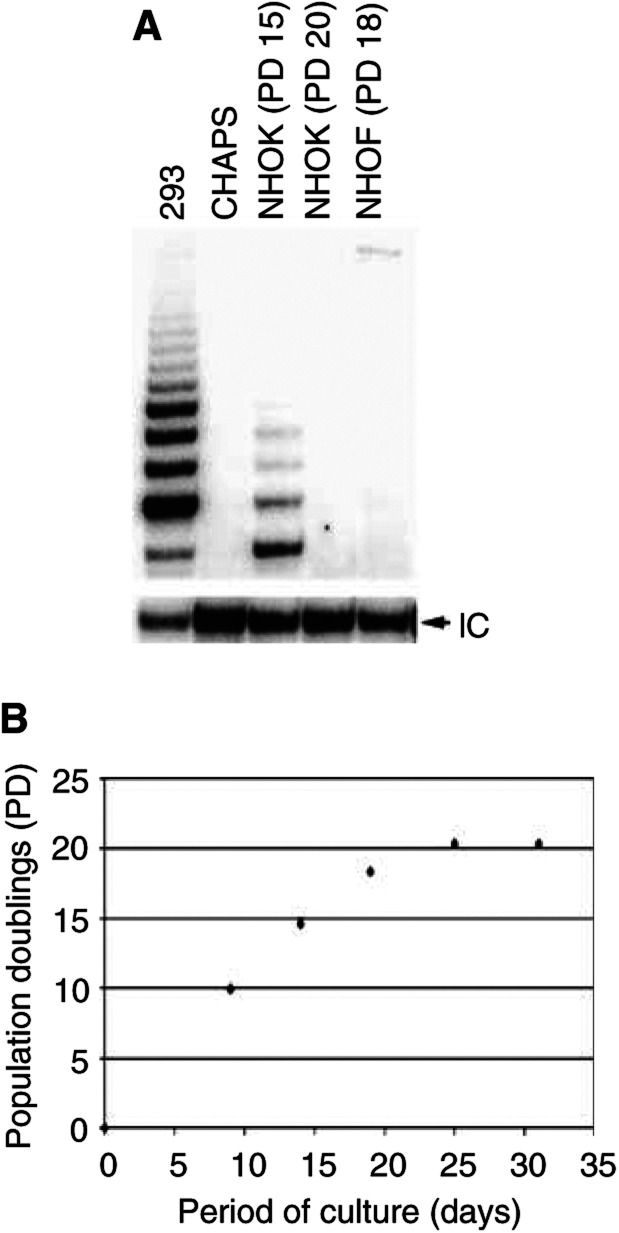
(**A**) TRAP assay of NHOK and NHOF. Cellular extracts obtained after different PD were tested for telomerase activity. The positive control was 293 (adenovirus-transformed human embryonic kidney cells). The CHAPS buffer was used as a negative control. IC is an internal control amplification product provided in the assay kit. We repeated this experiment three times with NHOF and NHOK cultures derived from different donors and obtained similar results (data not shown). (**B**) Replicative lifespan of NHOK. The cell PD were calculated as described in Materials and Methods. NHOK at PD 15 and PD 20 were used for the TRAP assay.

Our previous studies have shown the absence of telomerase activity in NHOF, its presence in exponentially replicating NHOK, and its gradual diminution during senescence in NHOK (Kang *et al*, 1998). This finding was confirmed in the present investigation, subjecting the exponentially replicating NHOF and NHOK, and senescent NHOK, to the telomeric repeat amplification (TRAP) assay (Figure 1A

). Telomerase activity was not detected in replicating NHOF or in senescing NHOK, but was detected in exponentially proliferating NHOK (Figure 1).

**Figure 2:**
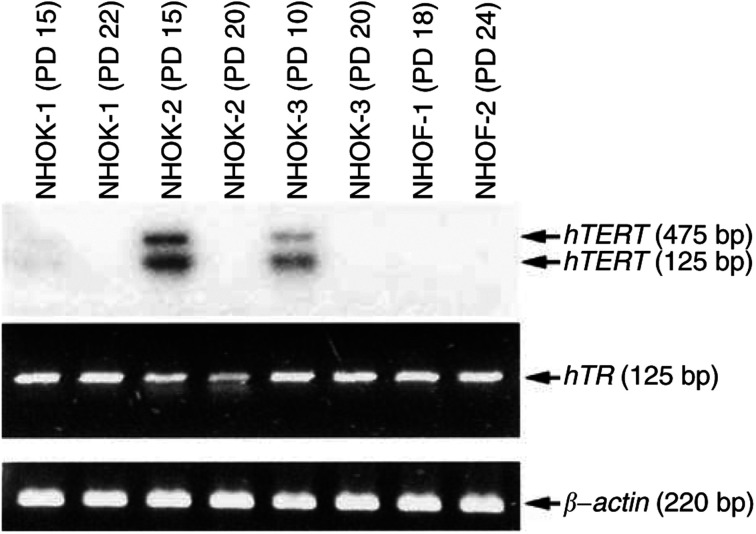
Expression of *hTERT* and *hTR* mRNA in NHOK and NHOF. Three different NHOK cultures (NHOK-1, NHOK-2, and NHOK-3) and two different NHOF cultures (NHOF-1 and NHOF-2) from various donors were examined in this experiment. The level of *hTERT* mRNA was detected by RT–PCR and subsequent Southern blotting with ^32^P-labelled full-length *hTERT* cDNA. A full-length (457 bp) and spliced isoform (275 bp) of *hTERT* mRNA was detected in rapidly replicating NHOK. The level of *hTR* mRNA was determined by RT–PCR. *β*-Actin mRNA was amplified as a control.

The regulation of telomerase activity by expression of the *hTERT* gene was confirmed. The expression of full-length and splicing variants of *hTERT* was also determined using primers flanking the RT domain of *hTERT* (Ding *et al*, 2002). The full-length *hTERT* mRNA containing the functional RT domain was detected in exponentially replicating NHOK as a 457 bp band (Figure 2

). The spliced variant of *hTERT* mRNA, also known as the *β*-deletion transcript containing a nonfunctional RT domain, was also identified in exponentially replicating NHOK as a 275 bp band (Figure 2). The *β*-deletion transcript causes a 183 bp deletion (bases 2342–2524) resulting in nonsense mutation, truncating the hTERT protein before the conserved RT motifs (Ulaner *et al*, 1998). *hTERT* mRNA was not detected in replicating NHOF or senescent NHOK which lacked telomerase activity (Figure 2). The presence of *hTR* mRNA in all tested cells indicated that *hTR* was not the limiting factor of the telomerase activity in NHOF and senescent NHOK (Figure 2).

### Transcriptional activity of an exogenous hTERT promoter in NHOF and NHOK

The absence of *hTERT* mRNA in NHOF and senescent NHOK could result from either one of the following forms of negative control: (1) repressive alteration in the *cis*-acting *hTERT* promoter region or (2) defect in *trans*-acting regulatory factors required for promoter activity. To distinguish between the two possibilities, we examined the transcriptional activity of an exogenous *hTERT* promoter in NHOF and NHOK. SCC-4, a telomerase-positive human oral cancer cell line, served as positive control (Kang *et al*, 1998). The pGL3B-TRTP luciferase reporter plasmid containing a full-length hTERT promoter (nucleotides −1665 to +5) was transiently transfected into cells. The luciferase activity of pGL3B-TRTP was measured and compared with that of the pGL3-control plasmid, which was driven by the SV40 enhancer/promoter and used to monitor the transfection efficiency (Horikawa *et al*, 1999; Takakura *et al*, 1999).

As shown in Table 1

**Table 1:** Exogenous *hTERT* promoter activity in NHOK, NHOF, and senescing NHOK

		**Luciferase activity (light units)^a^**		
**Cells**	**Telomerase activity**	**pGL3-basic (no promoter)**	**pGL3B-TRTP (hTERT promoter)**	**pGL3-control (SV40 promoter)**
SCC-4^b^	+	1.7±0.5 (0.03%)	610±61 (9.8%)^c^	6241±825 (100%)
NHOK (PD 10)	+	0.01±0.02 (0%)	244±13 (7%)	3507±113 (100%)
NHOK (PD 20)	−	0.004±0.002 (0%)	60.7±23 (6.9 %)	878±32 (100%)
NHOF (PD 24)	−	0.01±0.02 (0%)	115±4 (10.9%)	1059±148 (100%)

a Mean luciferase activity and standard deviation. The activity of pGL3-basic or pGL3B-TRTP was normalised with that of pGL3-control.

b SCC-4, telomerase-positive human squamous cell carcinoma cell line.

c Percentage of pGL3-control.

, the pGL3B-TRTP plasmid expressed luciferase activity, that is, 9.8% of the pGL3-control, in the telomerase-positive human oral cancer cell line SCC-4. The same level of activity was found in exponentially replicating (PD 10) and senescing (PD 20) NHOK (7.0 and 6.9%), respectively. A similar level of activity (10.9% of the pGL3-control) was detected in hTERT-negative NHOF. These findings indicate that the necessary factors for hTERT expression were present and active in NHOF and senescent NHOK. It also suggests that negative alteration of the *hTERT* promoter is involved in repression of endogenous hTERT expression.

### Methylation of the hTERT promoter in NHOF and senescent NHOK

Previous studies have shown that (1) promoter hypermethylation is frequently associated with gene silencing (Herman *et al*, 1999), (2) the telomerase gene has a typical CpG island domain for methylation in its promoter (Horikawa *et al*, 1999; Takakura *et al*, 1999), and (3) the human *hTERT* gene promoter is hypermethylated in cells with reduced telomerase activity (Devereux *et al*, 1999; Bechter *et al*, 2002). These findings suggested that the absence of *hTERT* gene expression in NHOF and senescent NHOK might be due to hypermethylation of the endogenous *hTERT* promoter. In the *hTERT* promoter, two CpG islands, the transcription start site and regions 3′ to the translation start site, have been well characterised (Cong *et al*, 1999; Horikawa *et al*, 1999; Takakura *et al*, 1999; Dessain *et al*, 2000). Therefore, it was possible to determine the presence of methylation in both CpG islands in NHOF and senescent NHOK by using bisulphite treatment of DNA and subsequent methylation-specific PCR (Dessain *et al*, 2000).

**Figure 3:**
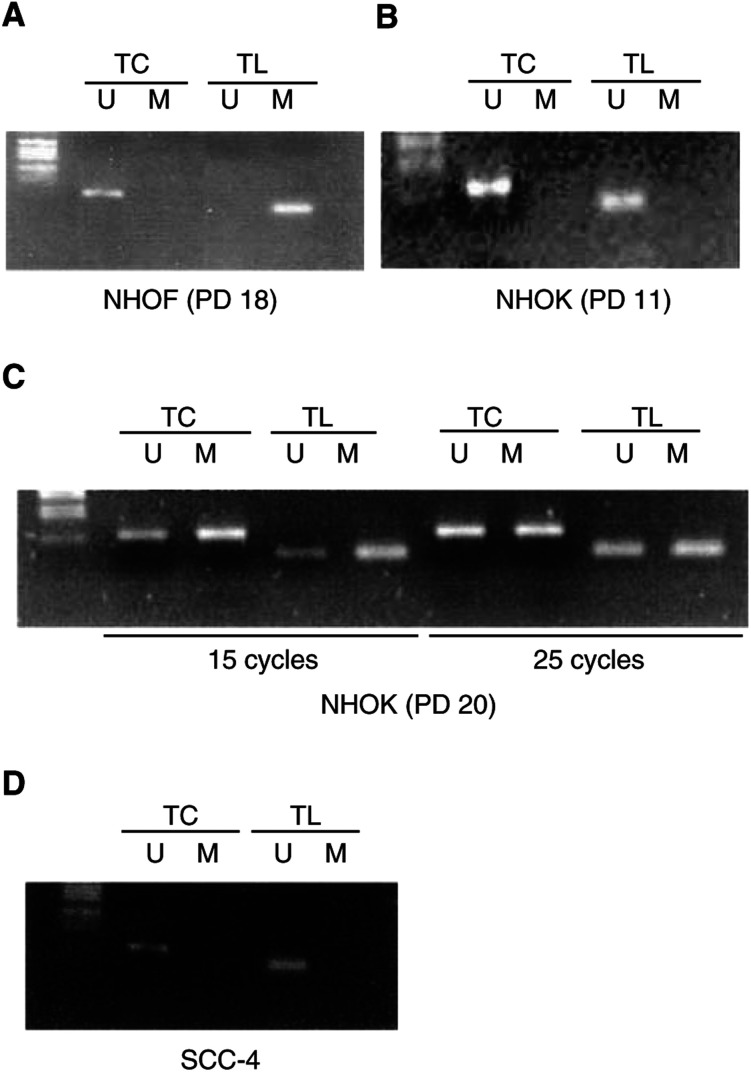
Methylation-specific PCR of the *hTERT* CpG islands in NHOF and NHOK. Genomic DNA was treated with bisulphite and tested with transcription start site (TC) and translation start site (TS) primer sets specific for either unmethylated (U) or methylated (M) hTERT promoter. (**A**) Methylation status of *hTERT* promoter in rapidly replicating NHOF (PD 18). The promoter was amplified with 25 cycles. (**B**) Methylation status of *hTERT* promoter in rapidly replicating NHOK (PD 15). The promoter was amplified with 25 cycles. (**C**) Methylation status of *hTERT* promoter in senescent NHOK (PD 20). The promoter was amplified with 15 or 25 cycles. (**D**) Methylation status of *hTERT* promoter in SCC-4. The promoter was amplified with 25 cycles.

In NHOF, as shown in Figure 3

, the region 3′ to the translation start site (TL) was completely methylated, while the region 3′ to the transcription start site (TC) was not methylated (Figure 3A). In rapidly proliferating NHOK (PD 11) that expressed *hTERT* mRNA and telomerase activity, both CpG islands in the *hTERT* promoter were hypomethylated (Figure 3B). Senescing NHOK (PD 20) showed methylation in both CpG islands (Figure 3C). With a reduced number of PCR cycles (15 cycles), the fraction of cells containing the methylated promoter was greater than that of cells containing the unmethylated promoter (Figure 3C). The methylation status of the hTERT promoter in the telomerase-positive cell line SCC-4 was also examined. Both CpG islands in the *hTERT* promoter were not methylated in the cell line (Figure 3D).

### Introduction of endogenous hTERT expression in NHOF and senescing NHOK exposed to 5-aza-CdR

**Figure 4:**
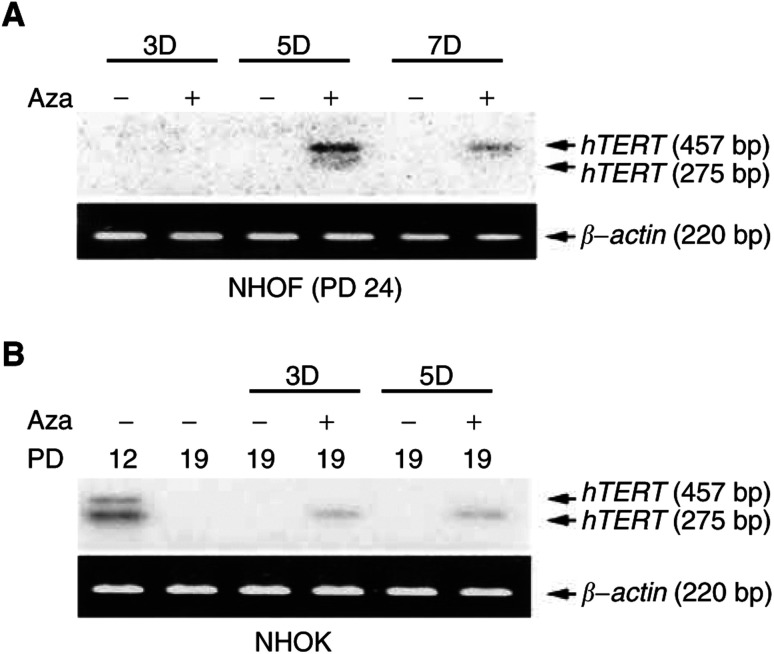
(**A**) Re-expression of endogenous *hTERT* mRNA in rapidly replicating NHOF (PD 24) exposed to 5-aza-CdR. (**B**) Re-expression of endogenous *hTERT* mRNA in senescent NHOK (PD 19) exposed to 5-aza-CdR. The cells were treated with 5 *μ*M of 5-aza-CdR for 3, 5, and 7 days and analysed for *hTERT* mRNA expression using RT–PCR Southern blotting. *β*-Actin mRNA was amplified as a control.

We investigated whether hypermethylation was solely responsible for repression of the *hTERT* gene expression by exposing the cells to 5-aza-CdR, the potent demethylating agent (Bender *et al*, 1998). The concentrations of 5-aza-CdR utilised in the present study were based on the studies reported by others (Devereux *et al*, 1999; Dessain *et al*, 2000). Furthermore, we determined the cytotoxic effect of 5-aza-CdR (at a range of 1–10 *μ*M) in exponentially proliferating NHOF and NHOK for up to 3, 7, or 10 days, and found that 1 or 5 *μ*M of 5-aza-CdR did not demonstrate any cytotoxic effect in these cells during the 7-day period, by determining the proliferation rate of cells as described elsewhere (Kang *et al*, 2000). NHOF and senescing NHOK were cultured in the absence or presence of 5 *μ*M 5-aza-CdR for 3, 5, and 7 days, and *hTERT* gene transcription was tested by RT–PCR Southern blotting (Figure 4

).

After 5 days of treatment, NHOF expressed a full-length (457 bp) and a spliced isoform (275 bp) of *hTERT* mRNA, whose expression was maintained during the entire 7-day treatment (Figure 4A). Senescing NHOK after 3 days of treatment with 5-aza-CdR re-expressed a full-length (457 bp) and a spliced variant (275 bp) of *hTERT* mRNA (Figure 4B). With longer exposure of the gel, the full-length hTERT mRNA was clearly visible in the 5-aza-CdR-treated NHOK (data not shown). Telomerase activity tested by the TRAP assay was not detected in NHOF and senescing NHOK exposed to 5-aza-CdR (data not shown). These results suggest that *hTERT* promoter hypermethylation in NHOF and senescent NHOK is linked to gene silencing, which can be reversed by demethylation.

## DISCUSSION

Activation of telomerase activity is thought to be a critical step in cellular immortalisation and transformation. Since telomerase activity is primarily regulated through *hTERT* expression, understanding of *hTERT* regulation in normal cells is crucial for the understanding of carcinogenesis. Our present studies provide evidence that methylation of the *hTERT* promoter is responsible for the repression of *hTERT* expression, which results in the lack of telomerase activity in NHOF and senescent NHOK.

Transcriptional activation has been considered a major mechanism for the regulation of the *hTERT* gene (Poole *et al*, 2001). Consistent with previous observations (Kim *et al*, 1994; Shay and Bacchetti, 1997), we found that expression of *hTERT* is a limiting factor for telomerase activity in NHOF and NHOK. Also, the presence of *hTERT* mRNA encoding functional or defective spliced isoforms has been demonstrated in several types of normal and pathogenic human cells (Ulaner *et al*, 1998, 2000; Liu *et al*, 1999). The splicing pattern of *hTERT* mRNA has been considered part of a regulatory mechanism for telomerase activity, but the details are unknown (Kilian *et al*, 1997; Ulaner *et al*, 1998, 2000; Wick *et al*, 1999). The *β*-deletion transcript causes premature termination of translation, resulting in truncation of the protein, and has been observed in various cell types (Nakamura *et al*, 1997; Wick *et al*, 1999; Ding *et al*, 2002). In the present study, we have detected the full-length and the *β*-deletion transcripts of *hTERT* in normal oral keratinocytes. Similar to our observations, coexpression of the full-length and the *β*-deletion transcripts have been reported in normal ovarian tissues (Ulaner *et al*, 2000) and human papillomavirus-immortalised endocervical cells (Ding *et al*, 2002). According to a previous report (Yi *et al*, 2001), a *β*-deletion splice variant was dominantly expressed (80–90% of total hTERT mRNA) in various human epithelial cells. Since NHOK is an epithelial cell, the domination of the *β*-deletion splice variant is not surprising. Although the reason for the dominant reactivation of the *β*-deletion splice variant upon the 5-aza-CdR treatment is not known, we could not rule out the possibility that the half-life (stability) of the *β*-deletion splice variant is longer than the full-length hTERT mRNA, and autosecretion of TGF-*β* retains high expression of the inactive *β*-deletion splice variant in NHOK culture (Cerezo *et al*, 2002).

After testing the transcriptional activity of an exogenous *hTERT* promoter in NHOF, rapidly proliferating NHOK and senescent NHOK, we found significant transcriptional activity in all tested cells, regardless of the presence of telomerase activity. The promoter region (nt –1665 to +5) examined in this study included the 59 bp region (nt −208 to −150) that was responsible for the maximal promoter activity (Horikawa *et al*, 1999). The data showed that in NHOF and senescing NHOK: (1) the transcriptional machinery necessary for *hTERT* promoter activity in replicating NHOF functioned as well as in NHOK-expressing *hTERT* mRNA and (2) the transcriptional machinery necessary for the *hTERT* promoter activity was not activated or inactivated during senescence in NHOK. Therefore, the absence of *hTERT* expression does not appear to be caused by a defective transcriptional machinery, but by an altered endogenous *hTERT* promoter.

In contrast with other reports of unmethylated *hTERT* promoters in normal fibroblasts (Devereux *et al*, 1999; Dessain *et al*, 2000), our studies show that *hTERT* expression appears to be correlated with the methylation status of the promoter in NHOF, suggesting that promoter methylation does provide a means to regulate *hTERT* expression in cells. To rule out the possibility of experimental errors and donor variation, we performed two assays in three strains of NHOF from different donors and obtained consistent reproducible results. Therefore, the hTERT promoter is tissue-specifically methylated and silenced in normal human fibroblasts (Imamura *et al*, 2001; Shiota *et al*, 2002).

Previously, we reported that replication arrest preceded differentiation in NHOK serially subcultured *in vitro* (Kang *et al*, 2000). The silencing of hTERT during squamous cell differentiation was demonstrated (Harle-Bachor and Boukamp, 1996). Based on these observations, we speculate that the silencing of hTERT due to promoter methylation is also in part associated with the silencing of hTERT during squamous differentiation *in vivo*.

Hypermethylation of the *hTERT* promoter during senescence in normal oral keratinocytes was correlated with the diminution of telomerase activity and *hTERT* mRNA expression. The involvement of promoter methylation in *hTERT* gene regulation during senescence in oral keratinocytes was further confirmed by induction of *hTERT* expression in senescent NHOK exposed to 5-aza-CdR. This finding supported the concept that the differentiation of cells is associated with changing methylation status at many loci in the genome (Shiota *et al*, 2002). While *hTERT* expression was restored by exposure to 5-aza-CdR, telomerase activity was not. Although the reason for inability of the chemical to restore telomerase activity is not known, we speculate that the inhibitory effect of 5-aza-CdR on DNA synthesis and telomerase activity may be the cause. 5-aza-CdR inhibited both DNA synthesis and telomerase activity in exponentially replicating NHOK (our unpublished data).
